# Molecular and cellular characteristics of human and non-human primate multipotent stromal cells from the amnion and bone marrow during long term culture

**DOI:** 10.1186/s13287-015-0146-6

**Published:** 2015-08-22

**Authors:** Olena Pogozhykh, Denys Pogozhykh, Anna-Lena Neehus, Andrea Hoffmann, Rainer Blasczyk, Thomas Müller

**Affiliations:** Institute for Transfusion Medicine, Hannover Medical School, Carl-Neuberg-Straße 1, 30625 Hannover, Germany; Department of Orthopaedic Surgery, Hannover Medical School, Anna-von-Borries-Straße 1-7, 30625 Hannover, Germany

## Abstract

**Introduction:**

Multipotent stromal cells (MSCs) are among the key candidates in regenerative medicine. However variety of MSC sources and general heterogeneity lead to controversial data in functional characterization. Furthermore, despite intensive usage as preclinical animal model, little is known about MSCs of the common marmoset monkey.

**Methods:**

MSCs derived from placental amnion and bone marrow samples from human and common marmoset were characterized in parallel over 12 passages to monitor similarities and significant differences (p ≤ 0.05, Student’s t-test) in MSC markers and major histocompatibility complex (MHC) class I expression by immunohistochemistry, flow cytometry, real-time PCR, metabolic activity test, with special focus on pluripotency associated genes.

**Results:**

Human and non-human primate MSCs were characterized for expression of MSC markers and capability of differentiation into mesenchymal lineages. MSCs could be cultured more than 100 days (26 passages), but metabolic activity was significantly enhanced in amnion vs. bone marrow MSCs. Interestingly, MHC class I expression is significantly reduced in amnion MSCs until passage 6 in human and marmoset, but not in bone marrow cells. For MSC markers, CD73 and CD105 levels remain unchanged in amnion MSCs and slightly decline in bone marrow at late passages; CD166 is significantly higher expressed in human MSCs, CD106 significantly lower vs. marmoset. All cultured MSCs showed pluripotency marker expression like Oct-4A at passage 3 significantly decreasing over time (passages 6–12) while Nanog expression was highest in human bone marrow MSCs. Furthermore, human MSCs demonstrated the highest Sox2 levels vs. marmoset, whereas the marmoset exhibited significantly higher Lin28A values. Bisulfite sequencing of the Oct-4 promoter region displayed fewer methylations of CpG islands in the marmoset vs. human.

**Conclusions:**

Little is known about MSC characteristics from the preclinical animal model common marmoset vs. human during long term culture. Studied human and common marmoset samples share many similar features such as most MSC markers and reduced MHC class I expression in amnion cells vs. bone marrow. Furthermore, pluripotency markers indicate in both species a subpopulation of MSCs with true ‘stemness’, which could explain their high proliferation capacity, though possessing differences between human and marmoset in Lin28A and Sox2 expression.

## Introduction

Multipotent stromal cells (MSCs) are among the key candidates in the broad perspective of application in the field of regenerative medicine, tissue engineering, and cell replacement therapy. This status is determined by their relative availability from various sources, high plasticity, and immunomodulatory properties. Unlike the other promising candidates, such as embryonic stem cells (ESCs), MSCs do not face ethical and legislative issues and do not have modified genotypes, as in the induced pluripotent stem cells (iPSCs), which makes MSCs more realistic for clinical usage in the near future. Among many varying definitions, MSCs are to date defined as a class of cells with the potential to self-renew and with certain “stemness” abilities, mostly to differentiate into multiple cell lineages within the same germ layer [1]. Furthermore, MSCs display a spindle-shaped morphology, are adherent to plastic, and are positive for certain surface markers, such as CD106^+^, CD105^+^, CD90^+^, CD73^+^, CD71^+^, CD44^+^, and CD29^+^, while being negative for hematopoietic markers, such as CD34^−^ and CD45^−^ [2, 3]. Yet marker combinations can vary depending on the variety of sources for isolation, resulting in a broad diversity and heterogeneity of obtained cell populations. Isolation of MSCs often implies invasive procedures and mostly does not result in large-scale numbers of cells. However, stromal cells of placenta and bone marrow, obtained by natural delivery and apheresis, provide one of the most reliable and abundant sources of MSCs [4]. Probably owing to the variety of MSC sources, as well as the heterogeneity of the derived cell populations in primary cultures, many controversial results exist from different groups in terms of various functions and the general characterization of MSCs [5–7]. Some authors question the proliferation capacities of placental MSCs compared with those from bone marrow, arguing that the placenta is a temporal organ with terminated postnatal function. Nevertheless, over the last decades placental MSCs have attracted attention in the field of research as well as in clinical application, which is determined by the virtual absence of ethical concerns and ease of access [8]. Furthermore, there are divergent reports about possible culture duration of MSCs [1, 6, 9] and changes in the expression of MSC markers and major histocompatibility complex (human leukocyte antigen (HLA)/MHC class I, which is a major obstacle in transplantation) over time [6, 10]. Lastly, the expression of pluripotency genes such as *Oct-4A*, *Nanog*, *Sox2*, *Klf4*, and *c-Myc* in MSCs is still discussed controversially [9, 11–14].

To address these topics, in this study we performed a comprehensive characterization of MSCs derived from human placental amnion and bone marrow over time in culture. Parallel to the human study, we utilized cells from our animal model, a small Brazilian nonhuman primate (common marmoset monkey, *Callithrix jacchus*), which is readily used as a preclinical model with the possibility of mimicking numerous pathologies, including various neurodegenerative and immune-relevant diseases, which are inherent to human and other primates [15]. While ESC and iPSC lines from *C. jacchus* are well established and characterized [16–19], little is known about the marmoset MSCs, especially in comparison with such cells of a human origin.

## Materials and methods

### Retrieval of tissue

In the case of the human, three samples of bone marrow were obtained during routine surgeries at Hannover Medical School in the Department of Orthopaedic Surgery, approved by the Ethical Commission of Hannover Medical School (ethic votum No. 565–2009). Five human placentas were donated anonymized after routine Caesarian-section birth in the Department of Gynecology at Hannover Medical School, Hannover, Germany and approved by the Ethical Commission of Hannover Medical School (ethic votum No. 2396–2014). All human samples were obtained in an anonymized form with consent from the patients. In the case of the nonhuman primate model, obtaining three samples of bone marrow (post mortem) and five samples of placenta (post natal) from healthy marmosets was approved by Institutional Animal Care from the Centre of Reproductive Medicine and Andrology (CeRA), Muenster, Germany. Placenta samples were collected after natural birth at CeRA.

### Isolation of marmoset bone marrow stromal cells

The bone marrow of marmoset was isolated post mortem by rupture of the tibia and femur of each animal immediately after death had been confirmed by a veterinarian. The cavity was flushed with a hypodermic needle attached to a syringe. For preventing coagulation and for cell singularization, a heparin phosphate-buffered saline (PBS) mix was utilized (5 IU/ml) and the bone marrow was separated by pipetting thoroughly for 3 minutes. After singularization, the cell suspension was transferred into red cell lysis buffer (NH_4_Cl 0.15 M, KHCO_3_ 10 mM, ethylenediaminetetraacetic acid (EDTA) 0.1 mM, pH 7.5) for 5 minutes and centrifuged at 200 × *g* for 10 minutes. The cell pellet was resuspended in the MSC culture medium consisting of Dulbecco’s Modified Eagle Medium (DMEM; Biochrom AG, Berlin, Germany), 15 % fetal calf serum (FCS; Biochrom AG), 1 % penicillin/streptomycin (Invitrogen GmbH, Karlsruhe, Germany), and 50 μM l-ascorbic acid-2-phosphate and plated on a cell culture dish (Greiner Bio-One GmbH, Frickenhausen, Germany).

### Isolation of human bone marrow stromal cells

Bone marrow aspirates were obtained by iliac crest aspiration during routine orthopedic procedures from three healthy donors. Human MSCs were isolated from fresh heparinized bone marrow aspirates by density gradient centrifugation and subsequent recovery of mononuclear cells. Cells were washed with PBS, then resuspended in MSC medium (DMEM; Biochrom AG), 10 % FCS Hyclone (Thermo Fisher Scientific, Schwerte, Germany), 1 % penicillin/streptomycin (Biochrom AG), and 2 ng/ml human recombinant fibroblast growth factor (FGF)-2 (PeproTech GmBH, Hamburg, Germany) and cultured at 37 °C with 5 % CO_2_ at 85 % humidity. Plastic-adherent cells were propagated as described previously [20].

### Cell isolation from placenta

Amnion membranes were washed with PBS with 10 % Ciprofloxacin (Fresenius Kabi, Bad Homburg, Germany), cut into small pieces, and incubated in the presence of 0.25 % trypsin for 1 hour at 37 °C. After trypsin digestion, samples were filtered through 100 μm cell strainer (BD Biosciences, Durhan, NC, USA), the cell suspension was centrifuged for 5 minutes at 300 × *g* (Heraeus Multifuge 1S-R; Thermo Fisher Scientific), and the cell pellet was resuspended in MSC growth medium and plated into 10 cm cell dishes (Cellstar; Greiner Bio-One GmbH). All cell samples were tested for mycoplasma contamination. With regard to the monoplacental nature of pregnancy, amniotic membranes from the marmoset were retrieved exactly around each umbilical cord to avoid a mixture of cells from different fetuses.

### Flow cytometry

After trypsinization and fixation in 4 % paraformaldehyde, cells were aliquoted into fluorescence-activated cell sorting (FACS) tubes and stained with antibodies. Cells incubated with secondary antibody were used as controls. Cells were incubated at room temperature with primary and secondary antibody for 1 hour respectively. After each step, cells were washed twice in PBS and then measured with a flow cytometer (FACSCalibur™; Becton Dickinson GmbH, Heidelberg, Germany) with a rate of 10,000 events per measurement. Cells incubated only with a secondary antibody were used as a negative control. BD CellQuest™ Pro software (version 6.0; Becton Dickinson GmbH) was used for analysis of the data with a regional statistics approach.

### Antibodies

Information on primary and secondary antibodies used for flow cytometry and immunohistochemistry (IHC) experiments is presented in Table 1.

**Table 1:** Antibodies used with corresponding working dilutions and origin

CD antigen	Company	Catalogue number	Dilution
Brachyury	Abcam (Cambridge, UK)	ab20680	1:100
CD90	Abcam (Cambridge, UK)	MRC OX-7 ab225	1:100
CD105	Dianova (Hamburg, Germany)	DLN-07243	1:100
CD73	Abcam (Cambridge, UK)	7G2 ab54217	1:250
CD 34	Beckman Coulter (Brea, CA, USA)	PN IM1167	1:50
Snail1	Santa Cruz (Dallas, TX, USA)	sc-28199	1:100
MHC class 1	AbD Serotec (Kidlington, UK)	MCA81	1:100
Secondary antibodies			
DyLight 488 donkey anti-mouse IgG	Dianova (Hamburg, Germany)	91518	1:400
DyLight 549 donkey anti-mouse IgG A	Dianova (Hamburg, Germany)	88693	1:400

MHC, major histocompatibility complex

### Immunohistochemical staining

For IHC staining, the Dako LSAB+System-HRP kit was used (Dako North America, Carpinteria, CA, USA). For the examination for mesenchymal markers, 2 × 10^4^ cells/well of each sample were seeded on a glass slide (20 mm in diameter) in a 12-well plate (Greiner Bio-One GmbH) and then fixed with 4 % paraformaldehyde after 24 hours. After washing in PBS, six drops of 3 % hydrogen peroxide were added to each well for 5 minutes to block endogenous peroxidase activities. The cells were incubated with respective biotinylated antibody overnight at 4 °C. The link solution was then applied to the glass slides for 30 minutes followed by streptavidin peroxidase for 30 minutes. The substrate solution was added to each well and incubated for 7–15 minutes until positive signals were visible on the glass slides. Then cells were washed twice with 1 ml distilled water. For a nuclei counterstaining, 300 μl hematoxylin were added and incubated for 3–5 minutes. After a last washing step with distilled water, the glass slides were transferred upside down onto an object plate in one drop of Mowiol® 4–88 (Sigma-Aldrich GmbH, Seelze, Germany) and dried overnight in the dark. Images were taken using a Keyence Biozero microscope (Keyence Germany GmbH, Neu-Isenburg, Germany).

### Total RNA isolation and RT-PCR

RNA was extracted using the peqGOLD Total RNA Kit (Peqlab GmbH, Erlangen, Germany). Briefly, after trypsinization the pellet was lysed in 400 μl RNA lysis buffer and transferred to a DNA removing column to remove contaminant DNA. After centrifugation at 12,000 × *g* for 1 minute, 400 μl of 70 % ethanol was added to the flowthrough. The lysate was loaded onto a Perfect Bind RNA Column and centrifuged at 10,000 × *g* for 1 minute to bind the RNA to the column. After a washing step with 500 μl RNA Wash Buffer I and washing twice with 600 μl RNA Wash Buffer II, the column was dried by centrifugation at 10,000 × *g* for 2 minutes. Finally, the RNA was eluted from the column by applying 50 μl sterile RNase-free dH_2_O. The RNA concentration was measured with a NanoDrop photometer ND-1000 (Thermo Scientific, Waltham, MA, USA). Extracted RNA was transcribed into cDNA using the High Capacity cDNA Reverse Transcription Kit (Life Technologies GmbH, Darmstadt, Germany). By adding Oligo(dT) primers (TIB Molbiol, Berlin, Germany) only the mRNA was transcribed. For the analysis of the mesenchymal mar-kers and immunorelevant molecules, a PCR reaction of 30 μl per sample was set up as follows: 24 μl dH_2_O, 3 μl of 1× PCR buffer (NEB, Frankfurt, Germany), 0.5 Units Taq polymerase (NEB), 100 mM dNTPs (Fermentas, St. Leon-Rot, Germany), 20 pmol/μl each primer, and 1 μg cDNA. Cycling conditions contained a precycling step at 95 °C for 3 minutes followed by 35 cycles of denaturation at 95 °C for 45 seconds, annealing at each corresponding primer temperature for 45 seconds, and extension at 72 °C for 90 seconds, with a final extension step at 72 °C for 10 minutes. In order to verify the product identity of the obtained cDNA fragments of the expected size, the sequencing performed in house utilizing a BigDye™ Terminator Cycle Sequencing Ready Reaction Kit (v1.1; PE Applied Biosystems, Waltham, MA, USA) according to the manufacturer’s instructions in 96-well PCR plates (Kisker, Steinfurt, Germany) in a C1000 Thermal Cycler (BioRad, Munich, Germany). A summary of oligonucleotide sequences, fragment sizes, and PCR conditions is presented in Table 2.

**Table 2:** Oligonucleotides with corresponding accession numbers

Gene	Sequence	Annealing temperature (°C)	Fragment size (base pairs)	Accession number
RPS29	5′-CGA AAA TTC GGC CAG GGT TC-3′	60	109	XM_009006002.1
	5′-TCG CGT ACT GAC GGA AAC AC-3′			
CD 90	5′-TCC CAG AAC GTC ACT GTG CT-3′	60	134	ENSCJAT00000027416
	5′-AGG GAT ATG AAA TCC GTG GC-3′			
CD 44	5′-TGG CCT TGG CTT TGA TTC TT-3′	60	73	ENSG00000026508
	5′-AGC TTT TTC TTC TGC CCA CA-3′			
CD 106	5′-TGG ATT CTG TGC CCA CAG AAA-3′	60	120	ENSCJAT00000006643
	5′-TGG TCA CAG AGC CAC CTT CT-3′			
CD 166	5′-ACG TGT TTG AGG CAC CTA CAA-3′	60	94	ENSG00000170017
	5′-AGC TGC TCT GTT TCG AGA AAT A-3′			
β2m	5′-CGA GAT GGC TAG CTC CGT G-3′	60	162	XM_002753411.2
	5′-GAT GGA TGA AAC CCA GAC AC-3′			
Oct 4 hs	5′-GGG TGG AGA GCA ACT CCG A-3′	60	123	NM_002701.5
	5′-GCA GAG CTT TGA TGT CCT GGG-3′			
Oct 4 cj	5′-GGG TGG AGA GCA ATT CCG A-3′	60	123	JQ627833
	5′-GCA GAG CTT TGA TGT CTT GGG-3′			
Sox 2	5′-ACA TGA ACG GCT GGA GCA A-3′	60	197	XM_002807565
	5′-GTA GGA CAT GCT GTA GGT GGG-3′			
Nanog	5′-AGC TGT GTG TAC TCA ATG AT-3′	60	121	ENSCJAT00000037278
	5′-TGG TTC TGG AAC CAG GTC TT-3′			
c-Myc	5′-AGC GAC TCT GAG GAG GAA CA-3′	60	150	XM_002759229
	5′-GCA CCT CTT GAG GAC CAG TG-3′			
Lin 28	5′-AGT GGT TCA ACG TGC GCA T-3′	60	181	XM_002751258
	5′-TCC AGA CCC TTG GCT GAC TT-3′			
Klf 4	5′-TTA ATG AGG CAG CCA CCT GG-3′	60	145	XM_002806507
	5′-AAG TCG CTT CAT GTG GGA GAG-3′			

### Real-time PCR

A 1 μl sample containing 10 ng cDNA was added to 19 μl SYBR Green master mix (Life Technologies GmbH) supplemented with respective forward and reverse primer concentrations optimized by prior titration and melting curve analysis for specificity and efficiency in triplicates. The quantitative PCR was performed using a StepOnePlus real-time platform (Life Technologies GmbH). Cycling conditions contained a precycling step at 95 °C for 10 minutes followed by 40 cycles of denaturation at 95 °C for 15 seconds and annealing at 60 °C for 1 minute. Additionally, melting curves were analyzed for the specificity of the products. In cluster comparison with other housekeeper genes, ribosomal housekeeper gene RPS29 was stable in our cells and tissues, and was therefore utilized for ΔCt normalization with additional control tissue skin for 2^–ΔΔCt^ analysis. Oligonucleotides were designed from a consensus sequence from human and marmoset utilizing the databases from the National Center for Biotechnology Information (NCBI) and the Wellcome Trust Sanger Institute/The European Bioinformatics Institute (Ensembl). Whenever possible, sequences from two different exons were used to exclude gDNA contaminations (Table 2).

### Cell proliferation assay (MTT test)

To evaluate the metabolic activity of human and marmoset MSCs through numerous passages in culture, we applied the Promega CellTiter 96® Non-Radioactive Cell Proliferation Assay (Promega, Madison, WI, USA). The cellular reduction of 3-(4.5-dimethylthiazol-2-yl)-2.5-diphenyltetrazoliumbromide (MTT) into formazan crystals is catalyzed by the mitochondria of living cells and is widely accepted as a marker for the growth and metabolic activity. The assay was performed according to the manufacturer’s specifications. In brief, 5 × 10^5^ cells of primary culture were seeded into 10 cm culture dishes and cultivated for 4 days until confluence with following repassaging. After each passage, cells were seeded at a density of 1 × 10^4^ MSCs/well of a 96-well plate (four parallels for each sample) and cultivated for 24 hours in 100 μl MSC medium (37 °C, 5 % CO_2_). After 24-hour incubation, 15 μl MTT reagent per well were added, incubated for 4 hours at 37 °C, and then 100 μl Stop Mix reagent was added to each well and incubated for 1 hour at room temperature in the absence of light. Afterwards, the formazan concentration was measured at 550–620 nm with an ELISA plate reader (BioRad 680). We compared the proliferation activities of all studied cell types with each other. The NIH 3T3 fibroblast immortal cell line was used as a control with a constant proliferation activity.

### Bisulfite sequencing assay

Evaluation of the methylation status of *Oct-3/4* promoter was performed by the bisulfite conversion method as described previously [21]. In brief, bisulfite treatment of genomic DNA converts unmethylated cytosines into uracil; respective changes were detected by PCR amplification followed by DNA sequencing.

Genomic DNA was produced with the peqGOLD Tissue DNA Kit (Peqlab BmbH) according to the manufacturer’s protocol, from which 200 ng were bisulfite converted with the EZ DNA Methylation-Direct™ Kit (Zymo Research, Freiburg, Germany). The fragment of interest was amplified from converted DNA by RT-PCR using ZymoTaq™ PreMix (Zymo Research), with oligonucleotides published previously [21]. Respective fragments were extracted from a 1.5 % agarose gel, ligated into pGEM-T Vector (Promega) and transformed into JM109 bacteria (Promega). Forty single-bacterium colonies of each sample were chosen; isolated plasmid DNA was submitted for sequencing (Eurofins Genomics, Ebersberg, Germany) and the obtained sequence was compared with unconverted genomic DNA with application of SnapGene software (GSL Biotech LLC, Chicago, IL, USA).

### Multilineage differentiation assays

The differentiation of MSCs into adipogenic, chondrogenic, and osteogenic lineages was performed by culturing cells in specialized culture media. In brief, all studied MSC samples were seeded with concentration of 5 × 10^4^ cells/well onto six-well culture dishes (Cellstar; Greiner Bio-One GmbH). Adipogenic differentiation of studied MSCs was induced by culturing the cells for 14 days in medium containing 1 μM dexamethasone (Sigma-Aldrich, St. Louis, MO, USA), 60 μM indomethacin (Sigma-Aldrich), 0.5 mM 3-isobutyl-1-methylxanthin (Sigma-Aldrich), 1 % (v/v) penicillin/streptomycin (Biochrom AG), and 10 μg/ml insulin (Sigma-Aldrich) in DMEM (Biochrom AG) supplemented with 20 mM HEPES zwitterionic buffer (Biochrom AG) and 20 % FCS HyClone™ (Thermo Fisher Scientific). After culturing the cells for 14 days, formation of lipid vacuoles was evaluated with Oil Red O staining. In brief, the cells were washed with PBS, fixed in 10 % formalin (Sigma-Aldrich) for 20 minutes, and rinsed twice with water followed by one final wash with 50 % ethanol (AppliChem GmbH, Darmstadt, Germany). Formed lipid vacuoles were detected by incubation for 10 minutes in Oil Red O (Sigma-Aldrich) in acetone/50 % ethanol (Merck KGaA, Darmstadt, Germany) and a final rinse with water. Adipogenic differentiation procedures for each sample were performed in triplicate and compared with undifferentiated cell control (*n* = 3) and visualized with a bright-field microscope (Keyence Biozero; Keyence, Osaka, Japan).

Osteogenic differentiation was performed by culturing cells for 21 days in medium containing 0.1 μM dexamethasone, 0.05 mM l-ascorbic acid-2-phosphate (Sigma-Aldrich), 1 % (v/v) penicillin/streptomycin, and 3 mM sodium dihydrogen phosphate monohydrate (Carl Roth GmbH, Karlsruhe, Germany) in DMEM LG (Biochrom AG) supplemented with 20 mM HEPES zwitterionic buffer and 10 % FCS HyClone™. After culturing the cells for 21 days, mineralization of MSCs differentiated into osteoblasts was detected by Von Kossa staining. In brief, mineralized cells were washed twice with PBS and fixed with 10 % formalin, and then washed once with PBS and twice with double-distilled water (ddH_2_O) followed by addition of 1 % silver nitrate (Riedel de Haen GmbH, Seelze, Germany). After that, the plate was exposed for 30 minutes to sunlight, washed with ddH_2_O, stained with 5 % sodium thiosulfate (Sigma-Aldrich) for 5 minutes, and rinsed again with ddH_2_O. Osteogenic differentiation procedures for each sample were performed in triplicate and compared with undifferentiated cell control (*n* = 3) and visualized with a bright-field microscope (Keyence Biozero).

For chondrogenic differentiation, 2.5 × 10^5^ cells/well were pelleted in V-shape 96-well plates (Cellstar; Greiner Bio-One Gmbh) by centrifugation at 200 × *g* for 5 minutes. The pellet was incubated for 21 days in chondrogenic differentiation medium composed of DMEM HG culture medium ((Biochrom AG) supplemented with 20 mM HEPES buffer with addition of 1 % (v/v) penicillin/streptomycin, 0.1 μM dexamethasone, 1 % (v/v) ITS Universal Cell Culture Supplement Premix (Becton Dickinson GmbH), 0.17 mM l-ascorbin acid-2-phosphate (Sigma-Aldrich), 1 mM sodium pyruvate (Biochrom AG), 0.35 mM l-proline (Biochrom AG), and 10 ng/ml transforming growth factor-β3 (PeproTech GmbH). The medium was changed every 3 days. After 3 weeks, the pellet was fixed with 10 % paraformaldehyde (Carl Roth GmbH), sectioned at 7 μm, and stained with Alcian blue (Sigma-Aldrich) as an indicator of sulfated glycosaminoglycan (sGAG)-rich extracellular matrix.

### Statistical analysis

Statistical analysis was conducted utilizing Microsoft Excel® 2010. Student’s *t* test was applied for comparing complete groups with *p* ≤0.05 values considered as statistically significant.

## Results

### Morphology and immunohistochemistry

**Fig. 1:**
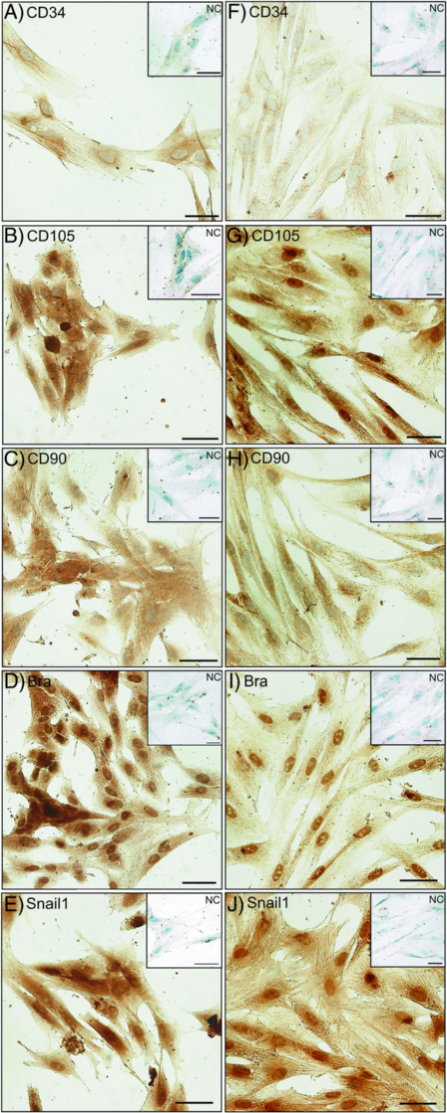
Typical MSC markers were detected in marmoset and human amnion MSCs. **a**–**e** Marmoset. **f**–**j** Human. Immunohistochemically negative signal for CD34^−^, but positive signal for CD105^+^, CD90^+^, Bra^+^, and Snail1^+^. *NC* absence of primary antibody

After three passages following the retrieval of primary culture, all of the cells displayed a typical adherent spindle-shaped fibroblast-like morphology forming a monolayer. Interestingly, in amnion samples of both species, some colony-like spots of high cellular density were observed. The antibodies against human antigens also reacted with marmoset samples in immunohistochemical staining displaying CD90^+^, CD105^+^, Snail1^+^, and Bra^+^, but CD34^−^ (Fig. 1a–j).

### Cellular proliferation capacity

**Fig. 2:**
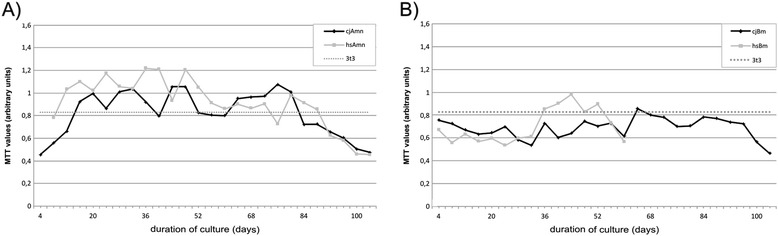
Evaluation of growth and metabolic activity of human and marmoset amnion and bone marrow-derived MSCs by the MTT test. Amnion-derived cells from both species have significantly higher proliferation potential (*p* = 0.0133) in long-term culture, whereas human bone marrow MSCs could not be expanded after day 60. *MTT* 3-(4,5-dimethylthiazol-2-yl)-2,5-diphenyltetrazolium bromide. Curve legends: **a** cjAmn - marmoset amnion, hsAmn - human amnion, 3t3 - control 3t3 cell line; **b** cjBm - marmoset bone marrow, hsBm - human bone marrow, 3t3 - control 3t3 cell line

When determining cellular viability and proliferation capacity via the MTT test, amnion MSCs from both species performed significantly better than bone marrow MSCs (*p* = 0.0133, Student’s *t* test) and also notably better than the immortalized NIH 3T3 cell line (Fig. 2a) which was used as a control owing to its stable proliferative abilities. Furthermore, human bone marrow cells stopped proliferation at 60 days of culture (Fig. 2b).

### Surface marker analysis

**Fig. 3:**
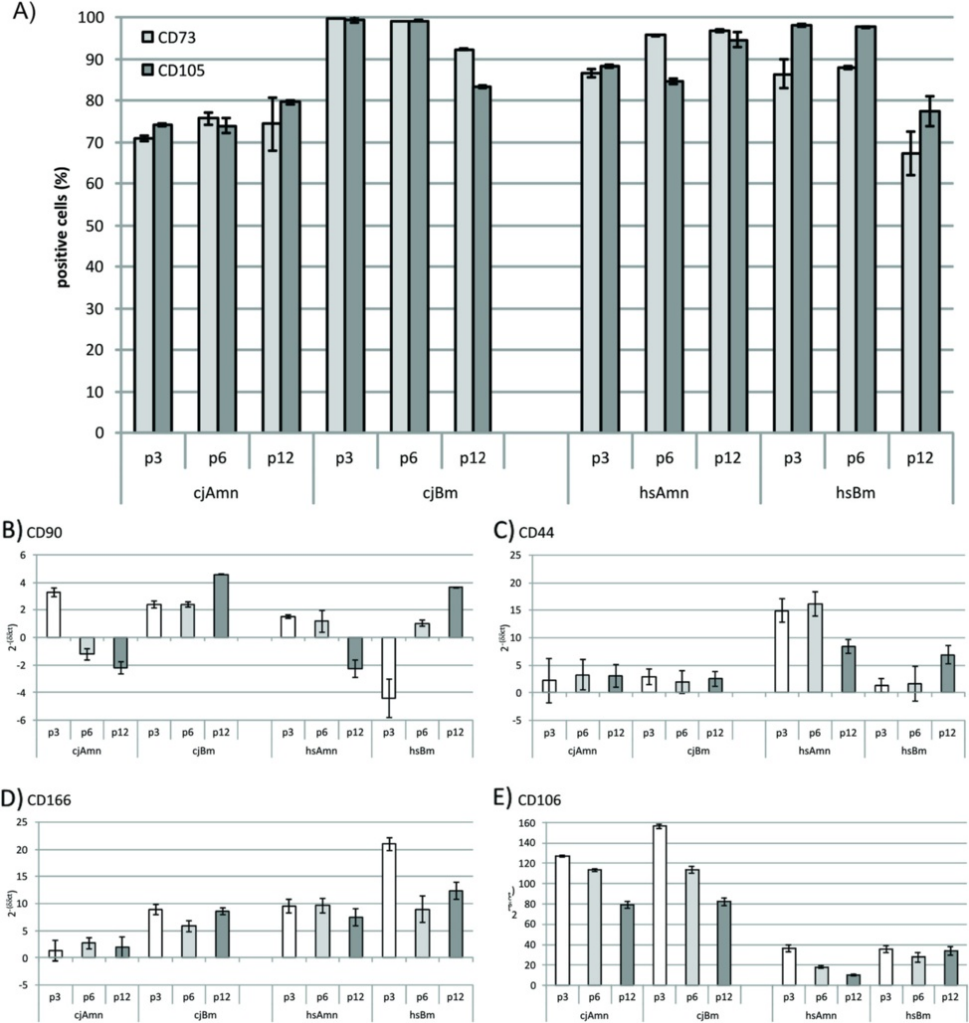
Analysis of typical mesenchymal markers. **a** Flow cytometry of CD73 and CD105. **b**–**e** Quantitative PCR of mesenchymal markers CD90, CD44, CD166, and CD106 (2^–ΔΔCt^ vs. skin). *Significant changes (Student’s *t* test, *p* ≤0.05)

Analysis by flow cytometry (Fig. 3a) showed that the number of CD73^+^ cells did not significantly change in marmoset amnion and bone marrow MSCs, 70.97 ± 0.54 at the early passage 3 compared with 74.43 ± 6.36 at the late passage 12, but significantly increased in human amnion MSCs from 86.54 ± 0.99 at the early passages to 96.78 ± 0.43 at passage 12. In human bone marrow samples, the number of CD73^+^ cells was 86.44 ± 3.44 at the early passages, significantly reducing to 67.4 ± 5.03 at passage 12. Furthermore, numbers of CD105-positive cells decreased significantly from passage 6 to passage 12 in the bone marrow samples of both species, from 99.26 ± 0.25 and 97.66 ± 1.19 to 83.46 ± 0.31 and 77.49 ± 3.51 in marmoset and human, respectively. In general, the number of CD105^+^ cells in marmoset was generally significantly lower than in human during all passages.

### Quantitative PCR results for MSC markers and MHC class I

In quantitative PCR, CD90 mRNA expression significantly decreased in all amnion MSC preparations during passaging (in marmoset, expression at the early passages was 3.27 ± 0.3-fold higher than in skin fibroblast control and at the late passages was downregulated to −2.20 ± 0.44-fold; in the human, expression was 1.51 ± 0.09-fold at passage 3 to −2.25 ± 0.62-fold at passage 12 respectively), but significantly increased in the bone marrow MSCs of both species (from 2.38 ± 0.18 at passage 3 to 4.56 ± 0.03 at passage 12 in marmoset, and from −4.41 ± 1.37 at passage 3 to 3.62 ± 0.06 at passage 12 in human respectively) (Fig. 3b). CD44 expression remained low and stable in marmoset amnion and bone marrow MSCs and in human bone marrow MSCs (Fig. 3c), but was significantly upregulated in human amnion cells (at passage 3 expression is 14.9 ± 2.10-fold, at passage 6 expression is 16.15 ± 2.20-fold, and at passage 12 expression is 8.35 ± 1.25-fold vs. skin). Activated leukocyte cell adhesion molecule (ALCAM, CD166) levels remained stable during passaging in all studied samples except the human bone marrow, where its expression was significantly higher compared with all other samples at passage 3, being 21.00 ± 1.21 (Fig. 3d). Interestingly, vascular cell adhesion molecule 1 (VCAM-1, CD106) expression was in general significantly higher in marmoset tissues vs. human. The expression level was 127.18 ± 0.94 at the early passages in marmoset amnion samples and 156.22 ± 1.99 in marmoset bone marrow samples at the early passages and was downregulated respectively to 79.22 ± 3.34 and 82.16 ± 4.12 at the late passages. In contrast, even the initial expression of CD106 in the human samples at the early passages was drastically lower than in marmoset samples, being 36.32 ± 3.05 for amnion MSCs and 35.42 ± 3.71 for bone marrow MSCs (Fig. 3e), with further reduction in later passages in amnion samples.

**Fig. 4:**
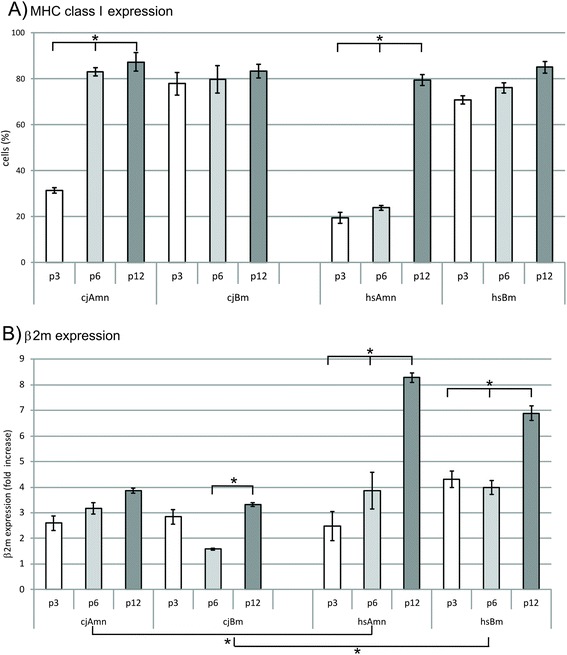
Evaluation of MHC class I expression by flow cytometry **a** and β2m by quantitative PCR **b** in MSCs. Amnion-derived cells from both species seem to regain immunogenicity triggered by MHC class I over time, whereas bone marrow MSCs already show full expression of MHC class I at passage 3. Interestingly, in marmoset expression of β2m is significantly lower than in human. *Significance by Student’s *t* test. *β2m* β2-microglobulin, *MHC* major histocompatibility complex

Interestingly, the levels of MHC class I expression (in case of the human also called HLA) at the early passages were significantly lower in the amnion samples of both species in comparison with bone marrow. Further, we detected a significant increase in positive amnion cells of both species over time by FACS analysis (from 19.42 ± 0.43 at passage 3 to 79.47 ± 2.0 at passage 12 in human, and from 31.35 ± 0.3 at passage 3 to 87.21 ± 4.0 at passage 12 in marmoset; Fig. 4a), whereas roughly 80 % of bone marrow cells were positive for W6/32 antibody from the beginning at passage 3 and remained so until passage 12 (Fig. 4a) in both species. This was in part reflected by quantitative PCR results detecting expression of beta-2-microglobulin (β2m) (Fig. 4b). Here, interestingly, marmoset expressed overall significantly less β2m molecules independent of tissue origin and passage when compared with human (Fig. 4b).

### Quantitative PCR of pluripotency genes and bisulfide sequencing

**Fig. 5:**
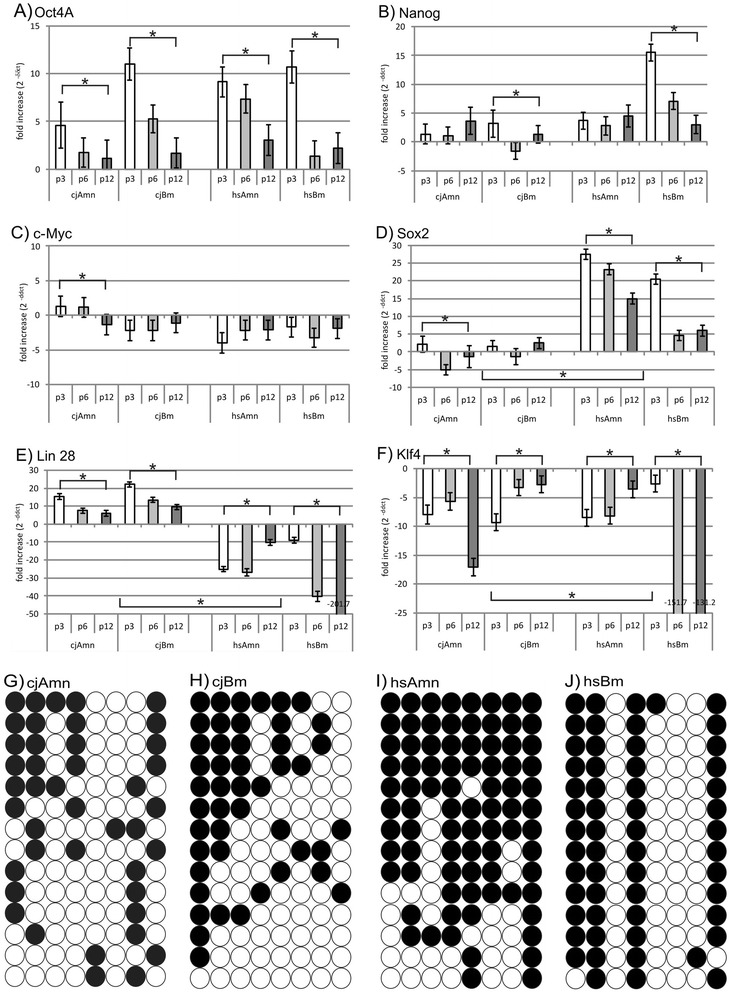
Analysis of typical pluripotency (PP) markers **a**–**f** and methylation of Oct-4 promoter **g**–**j**. Expression of PP markers could be confirmed (2^–ΔΔCt^ vs. skin), primarily in early passages **a, b**. In general, Oct-4A represented the highest levels **a**, whereas Klf4 levels were very low **f**. As expected, all pluripotency marker levels decrease over time. Interestingly, marmoset tissues display significantly lower Sox2 levels vs. human **f**, but also significantly higher Lin28A levels **e**. *Significant change (Student’s *t* test). The nature of all PCR products was confirmed by DNA sequencing. Methylation analysis of the Oct-4 promoter region revealed the heterogeneity within the cell populations: marmoset amnion MSCs appeared to contain the most cells with an unmethylated pattern (*open circles*) **g**, whereas human amnion cells contained more methylated (*black circles*) populations **i**

The existence of pluripotency genes in MSCs is controversially discussed and very carefully determined by quantitative PCR for typical pluripotency genes (Fig. 5a–f); additionally, bisulfite sequencing of the *Oct-4* promoter region was performed (Fig. 5g–j). In our experiments, *Oct-4A* mRNA expression levels decreased with passaging in all cell types. In marmoset amnion, at passage 3 *Oct-4A* expressed 4.60 ± 2.38 and at passage 12 was only 1.14 ± 1.92-fold higher than in the control; in human amnion, expression of *Oct-4A* is 9.16 ± 1.5 and decreases to 3.03 ± 1.59 at passage 12. The levels of expression of *Oct-4A* in bone marrow do not differ significantly between the species. They start at passage 3 with 11.02 ± 1.69 and 10.71 ± 1.67 and decrease at passage 12 to 1.68 ± 1.55 and 2.20 ± 1.60 in marmoset and human respectively. *Nanog* expression appeared to be at stably low yet detectable levels in both species’ amnion MSCs, but significantly decreased in human bone marrow MSCs from initial levels of 15.52 ± 1.46-fold vs. skin to 2.99 ± 1.6-fold (Fig. 5b). Interestingly, *c-Myc* expression was generally lower in all MSC types (Fig. 5c) than in controls (skin), although slightly upregulated at the early passages in marmoset amnion, whereas *Sox2* expression was significantly lower in marmoset MSCs than in human MSCs with 27.53 ± 1.43-fold expression vs. skin at the early passage 3 in human amnion, 20.4 ± 1.47 in early passage in the bone marrow, decreasing in both types of human MSCs significantly over time to 15.01 ± 1.57 and 5.98 ± 1.53 respectively (Fig. 5d) at the late passage 12. Complementary behavior was monitored for Lin28A, which was much higher expressed in marmoset than in human. The initial level of expression of Lin28A in marmoset amnion MSCs was 15.34 ± 1.53-fold and was downregulated to 6.04 ± 1.53-fold at passage 12; in bone marrow the initial level was 22.19 ± 1.45 and decreased to 9.65 ± 1.42 during passaging. In human samples, expression of Lin28A was significantly lower than in the skin fibroblast controls. *Klf4* levels were below skin controls in all cell types, further decreasing with passaging. An especially drastic decrease was observed in human bone marrow, with *Klf4* mRNA −2.61 ± 1.43 at passage 3 declining to −131.18 ± 1.45 at passage 12 (Fig. 5f). In all MSC preparations, a partially demethylated *Oct-4* promoter region could be detected (Fig. 5g–j). Marmoset MSCs showed in general fewer methylation sites than human MSCs.

### Differentiation experiments

**Fig. 6:**
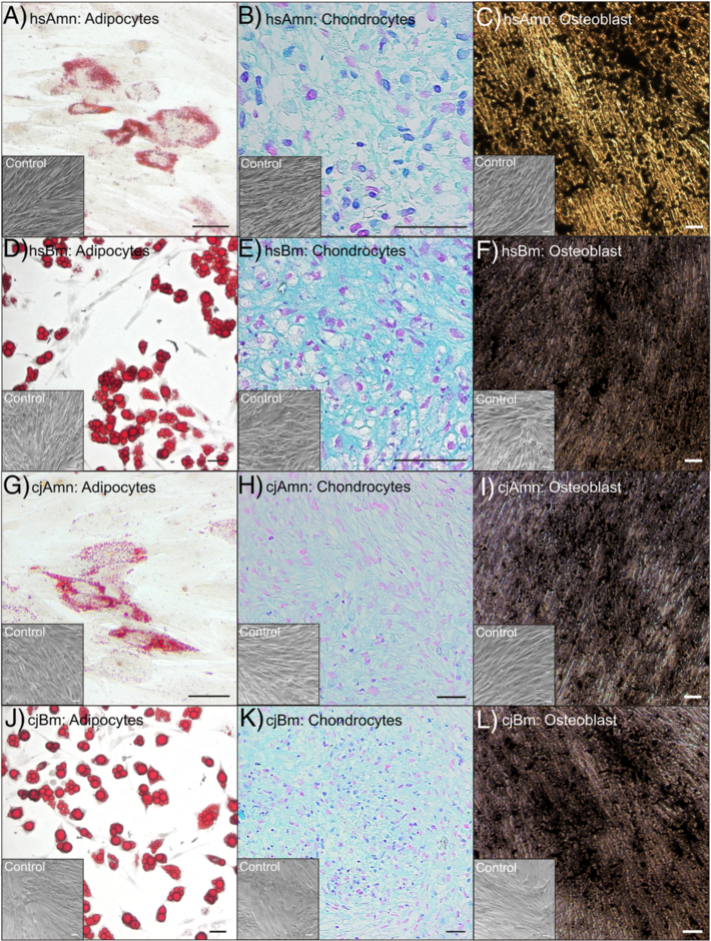
Differentiation potential of human and marmoset MSCs of different origin. MSCs were differentiated into adipogenic **a**, **d**, **g**, **j** (Oil Red O), chondrogenic **b**, **e**, **h**, **k** (Alcian Blue), and osteogenic **c**, **f**, **i**, **l** (Von Kossa) directions. Cells cultured in regular culture medium represent the negative control. During adipogenic differentiation, bone marrow samples of both species formed visually larger lipid vacuoles in comparison with amnion-derived cells **d**, **j**. Human amnion and human bone marrow samples showed higher visual intensity of Alcian blue staining **b**, **e**. Osteogenic potential was visually the same in all studied samples **c**, **f**, **i**, **l**. Scale bar = 50 μm

All studied samples from both species were capable of differentiation into adipogenic, osteogenic, and chondrogenic mesenchymal lineages (Fig. 6). Human bone marrow samples displayed the highest visual intensity of Alcian blue staining (Fig. 6e).

## Discussion

### Aim of the study

MSCs are the closest candidates for clinical application in regenerative medicine, tissue engineering, and cell replacement therapy. Their excellent availability from various sources, high plasticity, immunomodulatory properties, and genetic integrity make them an ideal cell type for clinical studies. Among these cells, bone marrow-derived, fat-derived, and placental MSCs are highly attractive, owing to availability of large-scale amounts of primary cell cultures from these sources. Unfortunately, owing to the variety of MSC sources and therefore heterogeneity of the obtained MSC populations in primary cultures, results from different research groups in terms of functions and general characterization of MSCs are controversial, especially with regard to pluripotency features of MSCs. Furthermore, little is known about the differences and similarities of MSCs of the common marmoset monkey in terms of surface markers and gene expression, a readily used preclinical nonhuman primate model in comparison with human. We therefore performed a systematic study with MSCs derived from the amnion of placenta and bone marrow from both species to clarify characteristics, similarities, and differences of these cells between human and the common marmoset.

### Comparison of morphology and differentiation potential

Cells from both species and both origins showed typical adherent spindle-shaped fibroblast-like morphology and were capable of differentiating into adipocytes, chondrocytes, and osteogenic direction; adipogenic potential seems to be enhanced in bone marrow vs. amnion MSCs in terms of vacuole size; and human bone marrow MSCs displayed the strongest Alcian blue staining. These observations could be explained by niche-specific differences.

### MSC immunohistochemistry

All multipotent MSC lines of both species and origins possessed typical marker combinations, CD44^+^, CD73^+^, CD90^+^, CD105^+^, CD106^+^, CD166^+^, Snail1^+^, and Bra^+^, but CD34^−^ as reported by other studies [2, 22, 23]. Other groups report more CD73^+^ and CD105^+^ MSCs found in the human bone marrow (roughly 98 %) vs. approximately 10–15 % lower numbers in placenta-derived primary cultures [6], which is in accordance with our data for human and marmoset. Various groups discuss controversial results on the presence or absence of Brachyury expression [22, 24]; in our case, both species and MSC types showed positive signals in IHC.

### Proliferation capacities

Our general duration of culture for amnion-derived MSCs over 120 days (26 passages) corresponds with results from other groups of 140 days with 36.9 ± 4.7 population doublings [24], and human placenta-derived MSCs have higher *ex vivo* proliferative capacity compared with human bone marrow [6, 25]. This is interesting from a biological point of view: the placenta itself is a temporary organ, which completed its physiological function at the moment of MSC isolation, but in our case the bone marrow MSCs ceased proliferation after day 60 in human. It could be speculated that such proliferation capacity in both species is derermined by the importance of placental functions for maintenance and development of the embryo in case of injuries.

### MSC characteristics by surface markers

CD73 and CD105 are generally accepted mesenchymal markers. CD73 (ecto-5′-nucleotidase) is additionally found on the surface of human T and B lymphocytes, facilitating in this case lymphocyte development and function [26]. CD105 (endoglin) is a type I homodimeric transmembrane glycoprotein and a key recognition structure in cellular adhesion, expressed primarily on vascular endothelial cells, chondrocytes, and syncytiotrophoblasts of term placenta, suggesting a critical role for endoglin in the binding of endothelial cells to integrins and other RGD receptors [27]. The number of CD73^+^ and CD105^+^ cells did not change significantly in long-term culture in both species and origins except for human bone marrow MSCs in passage 12 due to senescence. Other groups report that CD73 and CD105 levels in human bone marrow MSCs decrease significantly over time [28], which might be due to different long-term culture conditions.

### Quantitative PCR analysis of MSC markers

Additional commonly accepted MSC markers displayed similarities and differences between the species and cellular origins. CD90 (Thy-1), usually expressed in MSCs but also in hematopoietic stem cells and fibroblasts, neurons, and activated endothelial cells [27, 29], was slightly downregulated in amnion MSC samples and upregulated in bone marrow MSC samples of both studied species, which corresponds to the other results [30].

CD44 (hyaluronic acid receptor) is a cancer stem cell marker and a potential pluripotency marker involved in cell–matrix interaction, homing, adhesion, matrix assembly, and apoptosis resistance [27, 31]. CD44 expression was significantly elevated in human amnion cells, but was barely expressed in all other studied MSC samples of both species, which is in accordance with Kanda et al. [30] but was not confirmed by Wagner et al. [28]. We speculate that these differences are due to different methodological approaches (quantitative PCR vs. FACS).

CD166 is a widely used as a MSC marker with multiple functions such as cell–cell interactions, migration and homing, neural development, hematopoiesis, immune response, and tumor progression [27, 32]. In our study, CD166 levels are generally significantly higher in bone marrow MSCs due to their role in hematopoiesis, but in general significantly lower in the marmoset, which is a species-specific difference.

CD106 is a cell surface sialoglycoprotein connected with homing, migration, and adhesion of cultured cells [27, 33, 34] and a controversial history of presence and absence in the literature [9, 12]. In our study, levels of CD106 in the nonhuman primate were generally significantly higher in comparison with human samples, a species-specific difference which could be explained by the immunomodulatory role of this molecule in a naturally chimeric animal like *C. jacchus*.

### MHC class I expression

A major difference of bone marrow and amnion MSCs is their lack of MHC class I molecules on the surface, making them perfect candidates for potential clinical application owing to reduced immune responses during allogeneic transplantations [25, 35]. This potential could be confirmed in our study for human and marmoset, where all bone marrow MSCs displayed high MHC class I levels from passage 3 onwards. It should be mentioned that MSCs also display other immunosuppressing strategies than MHC class I suppression [36], such as suppression of CD4^+^ and CD8^+^ T-lymphocyte proliferation by the arrest anergy of T cells in the G0/G1 phase of the cell cycle [37]. Furthermore, and marmoset specific, we found a significant reduction of β2m molecules, which might be explained by the superior immune adaptation of the marmoset as a monoplacental animal with chimeric features. Some authors suggest that this tolerance is primarily due to tryptophan catabolizing enzyme indoleamine 2,3-dioxygenase (IDO) and HLA-G molecules binding to two major inhibitory natural killer (NK) receptors, killer-cell immunoglobulin-like receptors KIR1 and KIR2, thus inhibiting NK killing [25, 38, 39]. For the future it would be interesting to investigate whether bone marrow and amnion MSCs generally utilize different or synergistic strategies to evade the immune responses and whether the reduced β2m expression in the marmoset can also be found in other tissues.

### Presence of pluripotency markers

There is much debate about the presence or absence of pluripotency marker genes in MSCs in general [12, 14, 40–42]. In our study, the key player (*Oct-4A*) was clearly present during early passaging but decreased, as expected, over time in all MSC types. In accordance with this, bisulfite sequencing of the *Oct-4* promoter region showed high heterogeneity in primary cultures of all studied cell types. We speculate from these observations that there might exist a small population of cells with a tendency for pluripotency in primary MSC cultures decreasing rapidly owing to suboptimal culture conditions. *Sox2* and *Nanog* were expressed significantly higher in the placental samples in comparison with the bone marrow [9], whereas other groups showed decreased expression in middle and later passages of chorion MSCs [43]. In our study, detectable levels of *Nanog* were found in both human and nonhuman primate, remaining surprisingly constant over passaging except for the human bone marrow samples owing to the mentioned senescence.

Presence of *Sox2* is also reported controversially [43], but decreased in our study significantly over time in cells of both species and cellular origins. However, *Sox2* expression was significantly lower in marmoset MSCs than in human, which is not reflected in the literature and might be a specific difference between the species. Interestingly, Lin28A expression, a factor which is found in early embryogenesis, in primordial germ cells, in ESCs, and also in some adult tissues [44], was significantly higher in marmoset samples than in human. Presence of Lin28A was shown to have an impact on reprogramming efficiency in human and is subsequently required for the stable expansion of reprogrammed cells in human [45] and in marmoset [46] but is not expressed in human somatic cell lines [47]. *c-Myc* and *Klf4* induce transcriptional regulation and have a great impact on MSC differentiation. Additionally, *Klf4* has been shown to regulate MSC transcriptional activity and maintain cells in the undifferentiated state [48, 49]. In our experiments, *Klf4* levels were below skin controls in all cell types and also *c-Myc* expression was very weak in all MSC cultures.

In summary, indications of “stemness” were observed in early passages of both amnion and bone marrow-derived MSCs from human and marmoset. However, the expression level of *Sox2* was significantly lower and expression of Lin28A was significantly higher in the nonhuman primate.

## Conclusions

Application of stem cell therapy in treating various autoimmune and neurodegenerative diseases is rapidly progressing. Since the usage of highly pluripotent ESCs and iPSCs in clinics is exceedingly complicated by legislative, ethical, and safety issues, numerous studies are focused on application of MSCs and hematopoietic stem cells as a therapeutic tool being the most feasible approach for the purposes of regenerative medicine. However, adequate preclinical animal models are required to study long-term outcome in terms of graft rejection and efficiency of cellular therapies.

Our major findings in this manuscript are the following: human and marmoset cells share most common MSC features, and thus a small nonhuman primate common marmoset proves to be a valid preclinical model also in the field of MSC research; amnion MSCs of both species have higher proliferation activity in comparison with bone marrow MSCs, thus making them superior candidates for expansion; amnion MSCs of both species display low MHC class I expression in early passages, whereas bone marrow MSCs have significantly higher MHC class I levels from the very beginning, indicating a potential disadvantage in the case of transplantation; and all studied types of MSCs express certain levels of pluripotency markers, with differences in Lin28 and *Sox2* expression between the species. Taken together with findings on demethylated *Oct-4* promoter regions, we speculate that a small subpopulation of still pluripotent cells might be present in early passages. The developmental origin of these cells remains unclear and would require further investigation. In summary, we believe that the potential of placenta-derived MSCs is greatly underrated, particularly in terms of pluripotency and immunology. If this postulated pluripotent amnion cell subpopulation could be stabilized by cultural methods, the genetic and technical problems of virally induced pluripotent cells could be easily overcome in the future with a cell source that can be obtained noninvasively and is patient specific.

## Abbreviations

ALCAM: Activated leukocyte cell adhesion molecule
CD: Cluster of differentiation
CeRA: Centre of Reproductive Medicine and Andrology
ddH_2_O: Double-distilled water
DMEM: Dulbecco’s Modified Eagle Medium
EDTA: Ethylenediamine tetracetic acid
ELISA: Enzyme-linked immunosorbent assay
ESC: Embryonic stem cell
FACS: Fluorescence-activated cell sorting
FCS: Fetal calf serum
FGF: Fibroblast growth factor
HLA: Human leukocyte antigen
IDO: Indoleamine 2,3-dioxygenase
IHC: Immunohistochemistry
iPSC: Induced pluripotent stem cell
KIR: killer-cell immunoglobulin-like receptor
MHC: Major histocompatibility complex
MSC: Multipotent stromal cell
MTT: 3-(4,5-Dimethylthiazol-2-yl)-2,5-diphenyltetrazolium bromide
NK: Natural killer
PBS: Phosphate-buffered saline
sGAG: Sulfated glycosaminoglycan
VCAM: Vascular cell adhesion molecule
β2m: Beta-2-microglobulin
